# Continuous-Time Quantum Walk in Glued Trees: Localized State-Mediated Almost Perfect Quantum-State Transfer

**DOI:** 10.3390/e26060490

**Published:** 2024-06-02

**Authors:** Vincent Pouthier, Lucie Pepe, Saad Yalouz

**Affiliations:** 1 Institut UTINAM, Université de Franche-Comté, CNRS UMR 6213, 25030 Besançon, France; vincent.pouthier@univ-fcomte.fr; 2 Laboratoire de Chimie Quantique, Institut de Chimie, CNRS/Université de Strasbourg, 4 rue Blaise Pascal, 67000 Strasbourg, France; lucie.pepe@etu.unistra.fr

**Keywords:** quantum walk, quantum-state transfer, glued trees, complex networks

## Abstract

In this work, the dynamics of a quantum walker on glued trees is revisited to understand the influence of the architecture of the graph on the efficiency of the transfer between the two roots. Instead of considering regular binary trees, we focus our attention on leafier structures where each parent node could give rise to a larger number of children. Through extensive numerical simulations, we uncover a significant dependence of the transfer on the underlying graph architecture, particularly influenced by the branching rate (*M*) relative to the root degree (*N*). Our study reveals that the behavior of the walker is isomorphic to that of a particle moving on a finite-size chain. This chain exhibits defects that originate in the specific nature of both the roots and the leaves. Therefore, the energy spectrum of the chain showcases rich features, which lead to diverse regimes for the quantum-state transfer. Notably, the formation of quasi-degenerate localized states due to significant disparities between *M* and *N* triggers a localization process on the roots. Through analytical development, we demonstrate that these states play a crucial role in facilitating almost perfect quantum beats between the roots, thereby enhancing the transfer efficiency. Our findings offer valuable insights into the mechanisms governing quantum-state transfer on trees, with potential applications for the transfer of quantum information.

## 1. Introduction

Initially introduced by Farhi et al. [1], Continuous-time Quantum Walk (CTQW) is a pivotal paradigm in the development of quantum information theory [2,3,4,5]. From a theoretical point of view, CTQW serves as the quantum counterpart to the Classical Random Walk (CRW), a cornerstone concept in classical information theory [6,7,8,9,10]. In a CRW, a “walker” traverses the interconnected nodes of a complex network via a stochastic process, resulting in a diffusive motion. By contrast, CTQW unfolds a scenario where the walker behaves as a quantum entity evolving according to the Schrödinger equation. In this context, it has been demonstrated that the quantum laws governing the walker dynamics facilitate a coherent propagation across a complex network, leading to a novel form of transport that typically outperform CRWs [1,11].

The remarkable potential of CTQW has garnered interest across various scientific communities over the past decades. This concept has found diverse applications in quantum information theory, spanning both software and hardware domains. On the software side, CTQW has proven to be crucial for the development of various types of quantum algorithms. Notably, it has demonstrated superiority over classical walks in addressing questions such as the hitting time problem on complex graphs [1,11,12]. This superiority was particularly highlighted in the case of glued trees networks, where a quantum walker was shown to reach the right root from the initial left root exponentially faster than a classical walker [13,14]. Similarly, CTQW has also been instrumental in developing quantum computation methods for graph research [1,15,16,17,18] (analogous to Grover’s algorithm [19]), as well as for probing element distinctness [20], matrix product verification [21], and triangle finding [22], among other applications. Meanwhile, on the hardware side, CTQW has also emerged as a valuable tool for the study of the quantum transfer of information (or energy). In this context, the central issue is to find the keys leading to the realization of an efficient data transfer from one node to another in a given complex network. To tackle these investigations, many types of network topologies have been considered, including dendrimers [23,24,25], Apollonian networks [26], fractal networks [27,28], sequentially growing networks [23,24,25], and star graphs [29,30,31,32,33,34,35], among others. Such theoretical investigations were prompted by the possibility of manipulating genuine physical systems (e.g., spins, photons, or excitons) to realize physical CTQW. Noteworthy is that quantum experiments on either photonic or superconducting platforms were recently developed to corroborate some theoretical predictions of CTQW for the realization of an efficient quantum information transfer [36,37,38,39].

Prompted by the use of CTQW as a theoretical tool for hardware development, in the present work we focus on the question of quantum-state transfer on a specific family of networks: modular glued trees. Interestingly, several studies have already been realized on glued trees but mainly under the so-called binary architecture, i.e., when each parent node has only two children [13,14]. However, recent studies suggest that a change in the branching rate of these glued trees would lead to an increase in CTQW efficiency. Indeed, this feature has been observed experimentally in a photonic setup where heralded single photons were used as quantum walkers and laser-written waveguide arrays to simulate glued trees. By increasing the branching rate from two to five, it has been shown that the CTQW exhibits improved transport superiority over the CRW [40]. Similar features have been observed on two-fold Cayley trees with a branching rate equal to four [41]. In the present work, the dynamics of a quantum walker in glued trees is revisited by addressing the following question: what is the influence of the architecture of the trees on the efficiency of the quantum transfer between the two roots? Here, one introduces a two-parameter model (N,M) of modular s in which the degree of the roots *N* differs from the branching rate, *M*, of the other nodes. Therefore, depending on the model parameters, various dynamical regimes emerge due to the richness of the quantum walker’s eigenspectrum. In particular, it will be shown that the arising of a quantum superposition between states localized on the roots of the glued trees could favor near-perfect quantum transfer, a fundamental task in quantum information processing [42,43].

The present paper is organized as follows. In Section 2, the modular glued trees are described and the CTQW is defined. Next, one introduces the column subspace to map the CTQW to that of a one-dimensional chain, followed by the corresponding Hamiltonian. Finally, the ingredients needed to characterize the dynamics are described. The problem is investigated numerically in Section 3, where a detailed analysis of the transfer between the tree roots is carried out. Numerical results are finally discussed and interpreted in Section 4 based on analytical developments.

## 2. Theoretical Background

### 2.1. Glued Trees and CTQW

**Figure 1:**
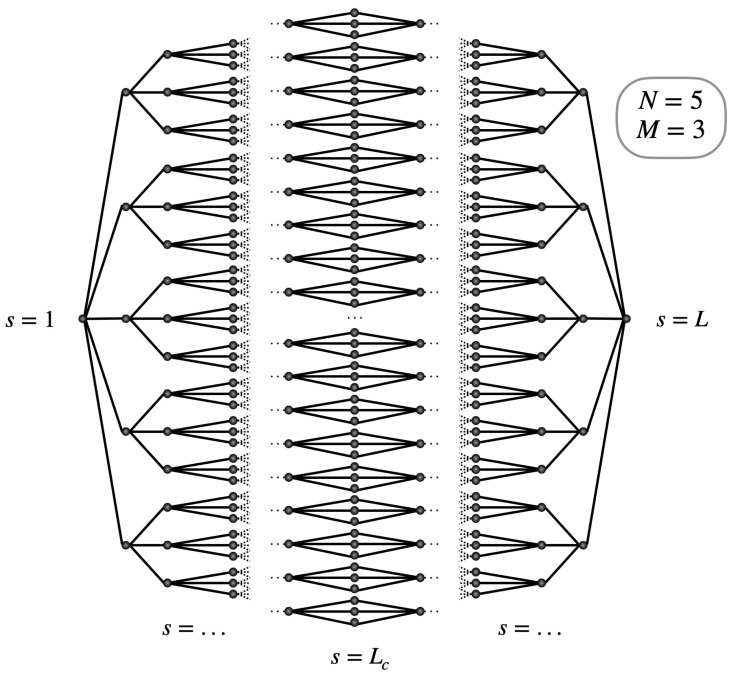
Representation of the glued tree $G_{NM}\left(L\right)$ for N=5 and M=3.

Throughout this paper, our attention will be directed towards modular glued trees, distinct from typical binary structures. Traditional binary trees adhere to a hierarchical arrangement where nodes are linked through parent–child relationships, with each parent node having a maximum of two children. In our study, we aim to explore more flexible structures where parent nodes can spawn a greater number of children, thus enhancing the adaptability of the tree. The resulting glued tree forms the graph $G_{NM}\left(L\right)$, illustrated in Figure 1, with parameters N=5 and M=3.

To describe the architecture of the modular glued trees, $G_{NM}\left(L\right)$, one introduces a column index s=1,2,…,L, with *L* being an odd integer. Let $L_{c}=\left(L+1\right)/2$ denote the central column. The columns $s=1,2,\ldots ,L_{c}$ characterize the leftmost tree whose root is specified by s=1. Conversely, the columns $s=L_{c},\ldots ,L$ refer to the rightmost tree whose root is identified by s=L. The two trees share the same leaves in the glued region, leaves which form the column $s=L_{c}$. The leftmost root s=1, whose degree is equal to $d_{1}=N$, is connected to *N* children, which form the column s=2. Each child of the column s=2 gives rise to *M* grandchildren, which form the column s=3. Therefore, the degree of each node in the column s=2 is equal to $d_{2}=M+1$. Similarly, each node of the second column is connected to *M* nodes that belong to the third column. Consequently, the degree of each node in the column s=3 is also equal to $d_{3}=M+1$. We continue this way until we reach column $s=L_{c}$, which contains the leaves. Each leaf being shared by the two glued trees, their degree reduces to $d_{L_{c}}=2$. Finally, starting from the leaves, we then go up the second tree until we reach the rightmost root s=L. The graph $G_{NM}\left(L\right)$ is thus symmetric with respect to the central column $s=L_{c}$ so that the degree, $d_{s}$, of the nodes of the *s*th column is defined as

(1) $$d_{s}=\left(M+1\right)\left(1-\delta_{s,1}-\delta_{s,L}-\delta_{s,L_{c}}\right)+N\left(\delta_{s,1}+\delta_{s,L}\right)+2\delta_{s,L_{c}}.$$

Each column *s* contains $M_{s}$ nodes labeled by the index $\ell =1,\ldots ,M_{s}$. The number of nodes by column is defined as

(2) $$M_{s}=\left(\delta_{s,1}+\delta_{s,L}\right)+NM^{L_{c}-2-\left|L_{c}-s\right|}\left(1-\delta_{s,1}-\delta_{s,L}\right).$$

On the glued trees $G_{NM}\left(L\right)$, we consider the motion of a quantum walker whose dynamics are described according to a standard CTQW [3,4,12,44,45,46]. Within this model, one associates a local state, |ℓ,s〉, to each node (ℓ,s). The set of states {|ℓ,s〉} provides a complete and orthonormal local basis for the Hilbert space of the walker. To describe the CTQW, different approaches have been introduced depending on the choice of Hamiltonian [47]. Here, we consider a CTQW generated by the Hamiltonian H=JΛ, where Λ is the Laplacian of the graph and where *J* denotes the hopping constant between the linked nodes [1,16]. Within the local basis, the Laplacian matrix is defined as

(3) $$\Lambda_{\ell s,\ell^{\prime }s^{\prime }}=\left\{\begin{array}{cc} -d_{s} & \mathrm{if}\;\left(\ell s\right)=\left(\ell^{\prime }s^{\prime }\right)\\ 1 & \mathrm{if}\;\left(\ell s\right)\;\mathrm{and}\;\left(\ell^{\prime }s^{\prime }\right)\;\mathrm{are}\;\mathrm{linked}\\ 0 & \mathrm{otherwise}. \end{array}.\right.$$

With these notations, the time evolution of the walker’s wavefunction on the graph’s site, $\psi_{\ell s}\left(t\right)$, is governed by the Schrodinger equation:

(4) $$i{\dot{\psi }}_{\ell s}\left(t\right)=J\sum_{\ell^{\prime }s^{\prime }}\Lambda_{\ell s,\ell^{\prime }s^{\prime }}\psi_{\ell^{\prime }s^{\prime }}\left(t\right).$$

To analyze the CTQW on the modular glued trees shown in Figure 1, one could opt to directly integrate the complete system of equations provided in Equation (4). However, in the present work a different approach will be employed due to the consideration of a specific initial condition, as explained in the following section.

### 2.2. Column Subspace and Restricted Hamiltonian

Our main objective here is to study the ability of the walker to traverse the network, i.e., to reach the rightmost root s=L, assuming that it initially started from the leftmost root s=1. In that case, the Schrödinger equation, Equation (4), can be expressed in a simpler way by mapping the problem onto a one-dimensional CTQW [13,14].

Indeed, readers can easily convince themselves that here the time evolution of the wave function $\psi_{11}$ of the leftmost root depends only on the sum of the wave functions of the second column s=2. In turn, the time evolution of this latter sum only depends on both the wave function $\psi_{11}$ and the sum of the wave functions on the third column s=3. Following this reasoning up to the rightmost root, it turns out that the Schrödinger equation simplifies by introducing the “column wave functions” as

(5) $$\psi_{s}=\frac{1}{\sqrt{M_{s}}}\sum_{\ell =1}^{M_{s}}\psi_{\ell s}.$$

Note that $\psi_{1}\equiv \psi_{11}$ and $\psi_{L}\equiv \psi_{1L}$ correspond to the walker wave functions on the left root and on the right root, respectively. Therefore, within this change of variables, the CTQW is finally described by a set of *L* coupled differential equations

(6) $$\begin{array}{c} i{\dot{\psi }}_{1}=-NJ\psi_{1}+\sqrt{N}J\psi_{2}\\ i{\dot{\psi }}_{2}=-\left(M+1\right)J\psi_{2}+\sqrt{N}J\psi_{1}+\sqrt{M}J\psi_{3}\\ i{\dot{\psi }}_{3}=-\left(M+1\right)J\psi_{3}+\sqrt{M}J\psi_{2}+\sqrt{M}J\psi_{4}\\ \ldots \\ i{\dot{\psi }}_{L_{c}}=-2J\psi_{L_{c}}+\sqrt{M}J\psi_{L_{c}-1}+\sqrt{M}J\psi_{L_{c}+1}\\ \ldots \\ i{\dot{\psi }}_{L-2}=-\left(M+1\right)J\psi_{L-2}+\sqrt{M}J\psi_{L-1}+\sqrt{M}J\psi_{L-3}\\ i{\dot{\psi }}_{L-1}=-\left(M+1\right)J\psi_{L-1}+\sqrt{N}J\psi_{L}+\sqrt{M}J\psi_{L-2}\\ i{\dot{\psi }}_{L}=-NJ\psi_{L}+\sqrt{N}J\psi_{L-1}. \end{array}$$

According to Equation (6), the dynamics of the column wave functions are governed by a Hamiltonian H, which is the restriction of the whole Hamiltonian *H* to the so-called column subspace [13,14]. This subspace is entirely generated by the set of *L* orthogonal column vectors |s〉, with s=1,…,L, defined as

(7) $$|s\rangle =\frac{1}{\sqrt{M_{s}}}\sum_{\ell =1}^{M_{s}}|\ell ,s\rangle .$$

With these notations, the column wave function $\psi_{s}\left(t\right)$ is the representation of the walker quantum state |ψ(t)〉 in the column basis, that is $\psi_{s}\left(t\right)=\langle s|\psi \left(t\right)\rangle$. The CTQW is thus generated by the Hamiltonian H expressed as

(8) $$\begin{array}{ccc} H= & - & \sum_{s=1}^{L}d_{s}J|s\rangle \langle s|+\sum_{s=2}^{L-2}\sqrt{M}J(|s\rangle \langle s+1|+|s+1\rangle \langle s|)\\ & + & \sqrt{N}J(|1\rangle \langle 2|+|2\rangle \langle 1|)+\sqrt{N}J(|L\rangle \langle L-1|+|L-1\rangle \langle L|). \end{array}$$

**Figure 2:**
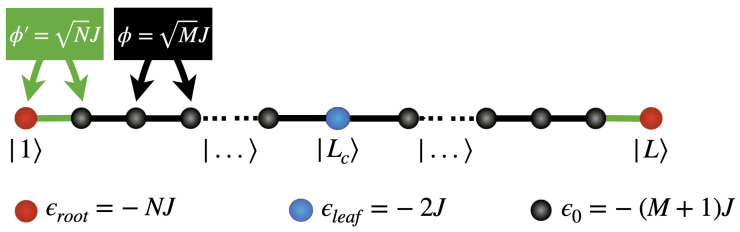
Graphical representation of the restricted Hamiltonian H to the column subspace. This Hamiltonian defines a tight-binding model on a finite-size chain. The nodes s=1 and s=L refer to the roots of the glued trees, whereas the nodes s=2,…,L−1 correspond to the column states.

As shown with Equation (8), the dynamics of the walker is isomorphic to that of a particle moving on a finite-size chain according to a standard tight-binding model. This chain, illustrated in Figure 2, involves the nodes s=1,…,L, associated with the states |1〉 (the walker is on the root s=1), |2〉 (the walker is uniformly delocalized over the column s=2), |3〉 (the walker is uniformly delocalized over the column s=3), …|L〉 (the walker is on the root s=L). In a general way, the nodes of the chain are characterized by a self-energy $\epsilon_{0}=-\left(M+1\right)J$, and the hopping constant between nearest neighbor nodes is $\Phi =\sqrt{M}J$. Nevertheless, the chain exhibits defects that originate in the singular nature of both the roots and the leaves of the glued trees. First, two energy defects are localized on the nodes s=1 and s=L, whose self-energy $\epsilon_{root}=-NJ$ is shifted from $\epsilon_{0}$ by an amount $\Delta_{r}=\left(M-N+1\right)J$. In addition, the hopping constant between s=1 and s=2, as well as between s=L and s=L−1, is equal to $\Phi^{\prime }=z\Phi$, with $z=\sqrt{N/M}$. Finally, an energetic defect is located on the central node $s=L_{c}$, whose self-energy $\epsilon_{leaf}=-2J$ is shifted by an amount $\Delta_{c}=\left(M-1\right)J$ when compared with $\epsilon_{0}$.

According to the standard properties of the tight-binding model [48,49], we expect the system to exhibit extended states that correspond to superpositions of forward and backward traveling waves whose energies belong to the allowed band $\left[\epsilon_{0}-2\Phi ,\epsilon_{0}+2\Phi \right]$. However, since the chain exhibits defects that break the symmetry of the problem, the Hamiltonian H supports additional states whose properties strongly differ from those of the traveling waves. We will show in the rest of the paper that such spectral richness favors the occurrence of specific CTQW. Note that, throughout the remainder of the article, the concept of “allowed band” will be used to underscore the deviations of our model from an ideal, uniform system. This perspective will enable us to better understand and appreciate the emergence of localized eigenstates that exist outside the typical energy range of an ideal, uniform chain. At this step, it is worth mentioning that several other studies have also focused on the impact of defects in the realization of continuous-time quantum walks in linear chains (for illustrative examples, see Refs. [50,51,52]).

### 2.3. Quantum Dynamics

By assuming that the walker is initially on the leftmost root s=1, its transport across the glued trees is described by the Hamiltonian H (Equation (8)). To simulate the associated dynamics, H is diagonalized numerically to determine the corresponding eigenvalues, $\left\{\epsilon_{\mu }\right\}$, and the associated eigenvectors, $\left\{|\phi_{\mu }\rangle \right\}$, labeled by the index μ=1,…,L. Consequently, one can compute the time evolution operator U(t)=exp(−iHt) written as

(9) $$U\left(t\right)=\sum_{\mu }\exp\left(-i\epsilon_{\mu }t\right)|\phi_{\mu }\rangle \langle \phi_{\mu }|.$$

From the knowledge of both the time evolution operator and the eigenstates, different observables can be computed. Here, special attention will be paid to characterizing the time evolution of the transfer probability $P_{L|1}\left(t\right)$, denoted by

$$P_{L|1}\left(t\right)={\left|\langle L|U\left(t\right)|1\rangle \right|}^{2}.$$

This probability measures the likelihood of a walker originating from the leftmost root of the glued tree to successfully traverse and arrive at the rightmost root at time *t*. Unveiling this probability provides fundamental information about the efficiency of quantum walker transport in the interconnected framework of glued trees.

## 3. Numerical Results

In this section, the previous formalism is applied to the description of the CTQW between the two roots of the glued trees. First, the spectral properties of the Hamiltonian H will be studied. Then, a detailed analysis of the walker’s dynamics will be presented, to assess its ability to traverse the graph and reach the rightmost root. Note that each simulation is carried out by considering the hopping constant *J* as the reference energy unit (i.e., J=1).

### 3.1. Spectral Properties of the Hamiltonian H

**Figure 3:**
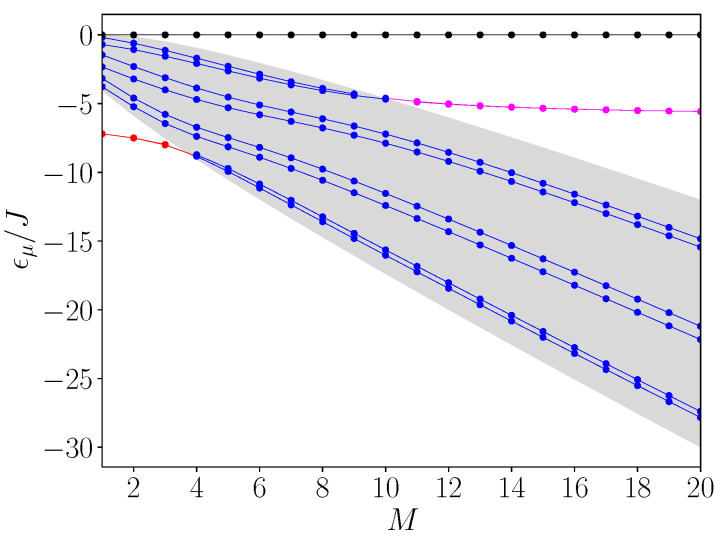
*M* dependence of the energy spectrum for L=9 and N=6. The gray zone defines the allowed band. Blue curves are used for eigenstates contained in the allowed band. These states are all spatially delocalized on the effective chain. Conversely, red, magenta, and black curves illustrate eigenstates outside the band that are spatially localized. For an illustration of the spatial delocalization of the eigenstates, see Figure 4.

In Figure 3, we illustrate the *M* dependence of the energy spectrum of the Hamiltonian H for L=9. The degree of the roots is fixed to N=6, whereas the branching rate of the “child” nodes varies from M=1 to M=20. The allowed band is defined by the gray zone. Figure 3 clearly shows the occurrence of specific states that lie outside the allowed band, and three different situations arise depending on the *M* values. Indeed, one first observes the existence of a unique state whose energy is equal to zero whatever *M* (see black curve). This state is always located above the allowed band provided that M>1. Note that for M=1, the zero energy corresponds exactly to the upper boundary of the allowed band.

**Figure 4:**
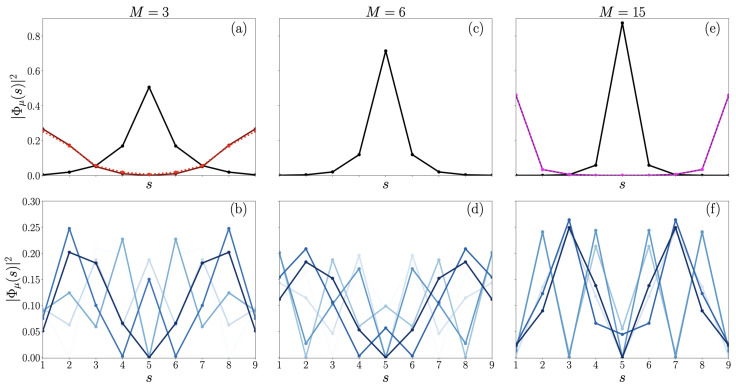
Eigenstates’ weights, $|\phi_{\mu }{\left(s\right)|}^{2}$, on the sites, *s*, of the chain. The three columns respectively represent the results obtained for three values of M=3, 6, and 15 (with fixed parameters L=9, N=6). The first row (**a**,**c**,**e**) illustrates the shape of the localized states detected, while the second row (**b**,**d**,**f**) showcases the delocalized eigenstates (in shades of blues). Regardless of the value of *M*, a zero-energy eigenstate localized at the center of the chain is always observed (see black curves in (**a**,**c**,**e**)). When M=3 in (**a**), two quasi-degenerate localized states are also present (shown in solid and dashed red curves), emerging below the allowed band. When M=15 in (**e**), two other quasi-degenerate localized states are also present (shown in solid and dashed magenta curves), emerging above the allowed band. Note that the spatial shape of the quasi-degenerate localized eigenstates almost superimpose (see red curves in (**a**) and magenta curves in (**e**)), which makes them hard to distinguish. Finally, the second row (**a**,**c**,**e**) shows that irrespective of the value of *M*, all complementary eigenstates in the allowed band exhibit spatial extension throughout the chain. As discussed in Section 3.2 and Section 3.3, these extended states may or may not strongly contribute to the dynamics, potentially leading to different quantum transport regimes.

Then, depending on the value of *M*, two other states can get out the allowed band. For instance, for M=3, in addition to the zero energy state, the spectrum supports two quasi-degenerated energy levels that lie below the allowed band (see red lines in Figure 3). The lower boundary of the band being equal to −7.46 J, the energies of the quasi-degenerated states are equal to −8.00 J and −7.97 J, respectively. In that case, the square modulus of the wave functions $|\phi_{\mu }{\left(s\right)|}^{2}$ on each site “*s*” of the effective chain is illustrated in Figure 4. As shown in Figure 4a, the zero energy level (black curve) corresponds to a state that is localized on the center of the chain $s=L_{c}$. The weight of the state on the central node is equal to 0.5. This state thus refers to a quite smooth localization of the walker around the central leaves of the glued trees. In marked contrast, the two low energy levels characterize states localized in the neighborhood of the side nodes s=1 and s=L (red solid and dotted lines). They thus refer to states localized on the roots of the glued trees. The weight of the states on the side nodes is approximately equal to 0.25. Note that detailed analysis of the wave functions reveals that these two states correspond to a symmetric and to an anti-symmetric superposition of two states localized on each root of the graph. Moreover, they oscillate from one node to another, indicating that the real part of their wave vector is equal to π (not drawn by considering the square modulus). Finally, as displayed in blue solid lines on Figure 4b, the remaining energy levels located inside the allowed band define extended states. They approximately correspond to the stationary waves that are observed in a confined environment. Note that these extended wave functions do not vanish on the nodes s=1 and s=L. They have almost the same weight on the sides of the chain, a weight approximately equal to 0.08.

As shown in both Figure 3 and Figure 4, a different situation arises for M=6. Of course, the zero energy level is still above the allowed band. It corresponds now to a state that is more strongly localized around the center of the chain (see Figure 4c), as its weight on the central node $s=L_{c}$ reaches 0.71. Conversely, all the other energy levels belong to the allowed band. Therefore, as illustrated in Figure 4d, they refer to extended stationary waves. The weight of the wave functions on s=1 and s=L now varies from one state to another over one order of magnitude. It approximately extends from 0.02 to 0.2.

Finally, for M=15 a different situation appears, as illustrated in both Figure 3 and Figure 4. One still recovers the zero energy level that remains above the allowed band, even if this latter now refers to a very strong localization on the center of the chain (black lines on Figure 4e), as its weight on $s=L_{c}$ is now equal to 0.87. In addition, the spectrum exhibits two quasi-degenerated energy levels that lie above the allowed band (solid and dotted magenta lines in Figure 3). The upper boundary of the band being equal to −8.25 J, the energies of the quasi-degenerated states are equal to −5.34 J and −5.33 J, respectively. Figure 4e reveals that these quasi-degenerate levels characterize states localized on the side nodes s=1 and s=L (magenta dotted lines). They thus refer to a strong localization on the roots of the glued trees, the weight of the states on the side nodes being approximately equal to 0.46. As for M=3, the study of the wave functions reveals that these two states correspond to symmetric and anti-symmetric superpositions of two states localized on each root of the graph (not observable in Figure 3 due to squared modulus).

### 3.2. Time Evolution of the Transfer Probability $P_{L|1}\left(t\right)$

Numerical simulations have been conducted to analyze the time evolution of the transfer probability $P_{L|1}\left(t\right)$. These simulations reveal that the transfer dynamics strongly depend on the branching parameter *M*, which is intimately associated with the presence (or absence) of localized states. As described in the previous sub-section, three main situations will also emerge depending on the *M*-parameter, and these will be detailed in the following paragraphs.

**Figure 5:**
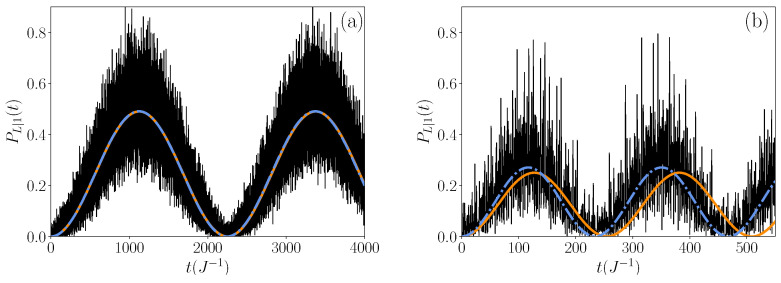
Time evolution of the transfer probability $P_{L|1}\left(t\right)$ for (**a**) M=2 and (**b**) M=3. The size of the chain is L=9 and the degree of the roots is N=6. Blue dashed lines define the smoothed probability $P_{L|1}\left(t\right)$ extracted from numerical calculations, while orange lines correspond to the theoretical expression given in Equation (28).

The first dynamical regime emerges when M<4, corresponding to the specific case where two spatially localized eigenstates exist below the allowed band (highlighted in red in Figure 3 and Figure 4). The transfer probability observed in this regime is illustrated in Figure 5a for M=2 and Figure 5b for M=3 (with fixed parameters L=9 and N=6). In Figure 5, we clearly observe that the time evolution of the probability $P_{L|1}\left(t\right)$ exhibits a rather singular pattern: a periodic slowly varying part of sine nature influenced by high-frequency noise. The long time period is approximately 2250 J^−1^ and 240 J^−1^ for M=2 (Figure 5a) and M=3 (Figure 5b), respectively, while the high-frequency noise evolves on a timescale of a few J^−1^. Based on this observation, the transfer probability could be roughly decomposed as a bi-partite signal:

(10) $$P_{L|1}\left(t\right)\approx P_{L|1}\left(t\right)+\Delta P\left(t\right),$$

where $P_{L|1}\left(t\right)$ represents the “smoothed probability”, corresponding to the periodic slow-varying part of the signal and ΔP(t) represents the high-frequency noise. Through numerical investigation, we observed that the long time period defining $P_{L|1}\left(t\right)$ follows the formula T=2π/Δϵ, where Δϵ is the difference between the energies of the two quasi-degenerate states below the allowed band. We then obtained an estimate of the smoothed probability as:

(11) $$P_{L|1}\left(t\right)\approx {\left|\sum_{\mu }^{\mathrm{Loc}.\;\mathrm{States}}\exp\left(-i\epsilon_{\mu }t\right)\langle L|\phi_{\mu }\rangle \langle \phi_{\mu }|1\rangle \right|}^{2},$$

resulting from a restriction of the time evolution operator (see Equation (9)) to only the two localized eigenstates present below the allowed band (highlighted in red in Figure 3 and Figure 4). The resulting signal $P_{L|1}\left(t\right)$ is represented with dashed blue lines in both panels of Figure 5. We observe that $P_{L|1}\left(t\right)$ accurately describes the averaged periodic behavior of the true signals over long time periods. However, the high-frequency noise plays a crucial role in interpreting the emergence of high transfer probability peaks in the exact transfer probability. For instance, in Figure 5a the first true maximum value of $P_{L|1}\left(t\right)$ is 0.95 (at t=990 J^−1^), while the smooth probability yields a maximum of $P_{L|1}\left(t\right)\approx 0.5$. Similarly, in Figure 5b the first true maximum value of the exact signal $P_{L|1}\left(t\right)$ is 0.81 (at t=118 J^−1^), while the maximum of the smooth probability is much lower, around $P_{L|1}\left(t\right)\approx 0.28$. In this dynamic regime, the high-frequency noise plays a significant role in the emergence of effective transfer from the left to the right root of the networks.

**Figure 6:**
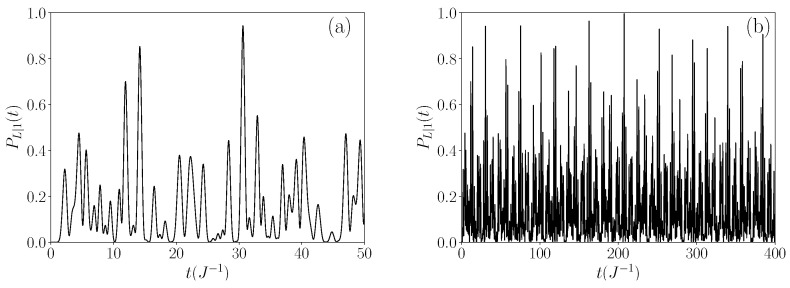
Time evolution of the transfer probability $P_{L|1}\left(t\right)$ for L=9 and N=M=6 over a timescale equal to (**a**) 50 J^−1^ and (**b**) 400 J^−1^.

The second type of regime occurs when 4≤M≤10, and no quasi-degenerate eigenstates extend beyond the allowed band. In this scenario, a completely different behavior emerges compared to the previous regime, as illustrated in Figure 6 for L=9, N=6, and M=6. Here, the time evolution of the probability $P_{L|1}\left(t\right)$ results from the coherent propagation of the walker, behaving like a wave packet undergoing multiple reflections at the roots of the glued trees. Initially zero, $P_{L|1}\left(t\right)$ exhibits a first peak at time t=2.2 J^−1^ (see Figure 6a), signifying the direct propagation of the walker from the leftmost root to the rightmost root. According to the properties of the tight-binding model, the walker has a group velocity approximately equal to $v\approx \sqrt{2}\Phi$ [53]. With $\Phi =\sqrt{M}J$, the time required to go from one root to another is τ≈(L−1)/v≈2.3 J^−1^, in reasonable agreement with the observations. As time progresses, the walker oscillates between the two roots, resulting in the emergence of a series of peaks. However, the amplitude of these peaks is not unity due to several influencing factors. First, dispersion causes the initial wave packet to irreversibly spread out. Second, the chain possesses defects leading to reflection/transmission processes, thereby introducing additional peaks. As depicted in Figure 6b, over a longer time scale the probability does not converge but exhibits a series of peaks, most of which have an amplitude smaller than or close to 0.4. Nevertheless, the figure distinctly showcases the occurrence of intense peaks distributed almost periodically. These peaks denote quantum recurrences that occur at specific revival times [53,54,55,56,57,58]. Typically, seven peaks have amplitudes larger than 0.9. Notably, on the considered time scale, $P_{L|1}\left(t\right)$ reaches a maximum value of 0.9973 at t=207.55 J^−1^. At this instance, a perfect transfer of the walker between the two roots of the glued trees becomes apparent.

**Figure 7:**
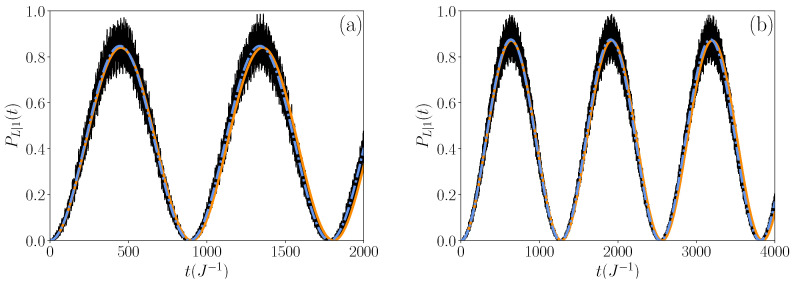
Time evolution of the transfer probability $P_{L|1}\left(t\right)$ for (**a**) M=15, (**b**) M=16. The size of the chain is L=9 and the degree of the roots is N=6. Blue dashed lines define the smoothed probability $P_{L|1}\left(t\right)$ extracted from numerical calculations. Orange lines correspond to the theoretical expression given in Equation (28).

The last observable dynamical regime arises when M>10, corresponding to the scenario where two spatially localized eigenstates emerge at the top of the allowed band (depicted in magenta in Figure 3 and Figure 4). The time evolution of the probability $P_{L|1}\left(t\right)$ is depicted in Figure 7 for M=15 and 16 (with L=9, N=6). Here, we observe that $P_{L|1}\left(t\right)$ can be decomposed as a bi-partite signal following Equation (10), such as the behavior observed when M<4 (refer to Figure 5). Specifically, $P_{L|1}\left(t\right)$ follows a slowly varying smoothed probability $P_{L|1}\left(t\right)$ that evolves almost periodically with time, as indicated by the blue dashed lines (note that $P_{L|1}\left(t\right)$ is numerically constructed from the two localized eigenstates at the top of the allowed band). The corresponding period is approximately T=890 J^−1^ and T=1250 J^−1^ for M=15 and M=16, respectively. $P_{L|1}\left(t\right)$ exhibits a high-frequency modulation varying over a timescale of a few $J^{-1}$. However, in contrast to the regime when M<4 (see Figure 5), the amplitude of this modulation is relatively small. Consequently, the main characteristics of the probability are very well captured by the behavior of the smoothed probability. Indeed, in contrast to the two previous regimes observed, the transfer probability remains significant over a wide timescale. For instance, between t=400 J^−1^ and t=500 J^−1^, the smoothed probability, representing the averaged signal, is 0.84. This extended duration of significant probability values could be particularly advantageous for generating efficient quantum transfer between the two tree roots, enabling better measurement control over a large time window. Similar characteristics emerge when M=16, as depicted in Figure 7b. Here, the maximum of $P_{L|1}\left(t\right)$ reaches 0.87 at around t=640 J^−1^.

### 3.3. Spectral Decomposition of the Initial Walker’s State

**Figure 8:**
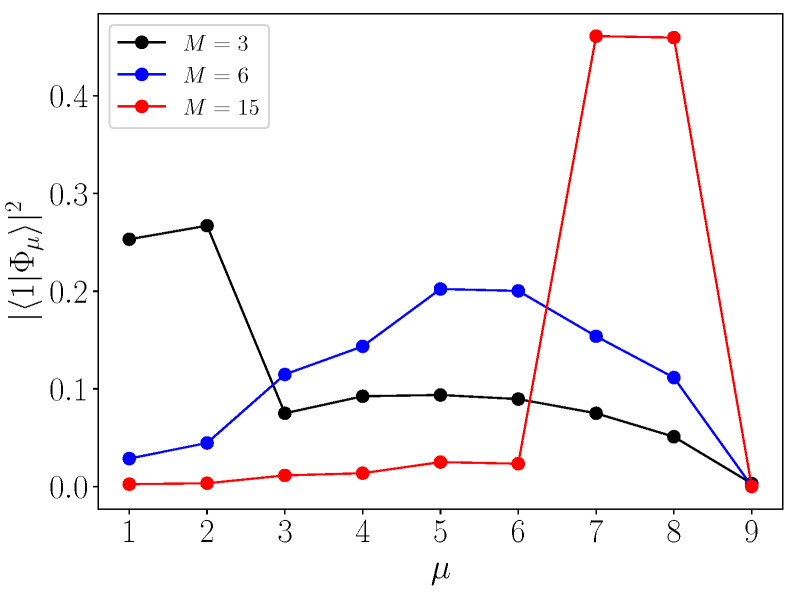
Spectral decomposition of the walker’s initial state for the three different dynamical regimes observed with M=3 (black curve), M=6 (blue curve), and M=15 (red curve). The parameters are L=9 and N=6.

To understand the three distinct CTQW regimes previously identified, we have examined how the walker’s initial state decomposes onto the eigenstates in each scenario. The results, depicted in Figure 8, show the decomposition for M=3 (localized degenerate eigenstates below the allowed band) in black, M=6 (no localized degenerate eigenstates) in blue, and M=15 (localized degenerate eigenstates above the allowed band) in red.

Starting with M=3, Figure 8 shows that the initial state primarily decomposes onto the two low-lying eigenstates indexed as μ=1 and μ=2, with a weight of 0.26, followed by a nearly uniform distribution across the remaining eigenstates (excluding the zero-energy state μ=9). This aligns with our observations regarding the first dynamical regime, where the significant portion of the transfer probability signal is carried by the two eigenstates localized on the network’s roots, as indicated by the smoothed probability (see Equation (10)). However, all other eigenstates also contribute significantly to the transfer, explaining the larger fluctuations observed around the corresponding smoothed probability, as evidenced in Figure 5.

Moving to M=6, where no degenerate eigenstates extend beyond the allowed band, Figure 8 demonstrates that the eigenstates contributing most to the transfer are localized in the middle of the band. Six eigenstates exhibit the highest weights, all falling within the interval [0.1,0.25]. Unlike the previous regime, here the dynamics are supported by a greater number of eigenstates playing similar roles. These characteristics elucidate why the transfer probability evolves erratically over time and cannot be decomposed into slow and fast-varying components in this case, as seen in Figure 6.

Finally, considering the last dynamical regime at M=15 where two eigenstates localized on the roots emerge above the allowed band (as illustrated in Figure 7), we observe that these two eigenstates, indexed as μ=7 and μ=8, carry the highest weights, nearly 0.5, implying that the initial state predominantly decomposes onto them. The remaining eigenstates play a very minor role, as indicated by their weights consistently below 0.03. Consequently, the walker’s dynamics reflect those of a two-level system, explaining the near-perfect quantum beats observed between the two roots of the networks in Figure 7.

### 3.4. Characterization of the Smoothed Probability and Optimization of the Transfer

The previous observations, conducted for fixed values of *L* and *N*, suggest that the emergence of localized eigenstates could facilitate the realization of more efficient and controlled quantum transfer across the network. More precisely, it appears that when localized eigenstates arise above the allowed band, they lead to a more robust transfer that exhibits a very high amplitude of smoothed probability. This is in contrast with the case where the dominant eigenstates are below the allowed band, resulting in a lower amplitude of smoothed probability. These features were evidenced for fixed values of *N* and *L*, and we will now demonstrate that they persist when varying these parameters.

**Figure 9:**
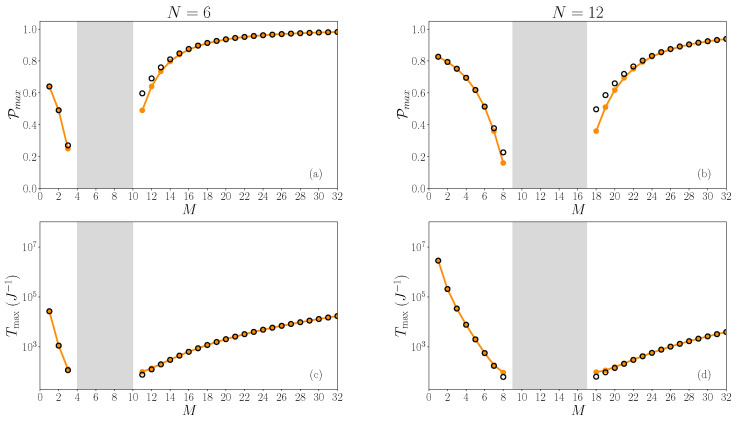
First row (**a**,**b**): illustration of the *M* dependence of the smoothed probability P. Second row (**c**,**d**): illustration of the associated period $T_{max}$. Left and right columns respectively display the results for N=6 (**a**,**c**) and N=12 (**b**,**d**). Black symbols correspond to numerical calculations, whereas orange symbols refer to theoretical estimates (as given in Equation (28)). The size of the graph is L=9. Gray areas correspond to the parameter-space where no states are localized outside the allowed band, except for the zero-energy state.

To highlight this feature, Figure 9 showcases the *M* dependence of the maximum value of the smoothed probability $P_{max}$ (first row) and the associated time $T_{max}$ (second row) for which this maximum arises. The left and right columns respectively show the results obtained for two different values of N=6 and N=12 (with a fixed size of the graph L=9). Black circles correspond to numerical calculations, whereas orange curves refer to theoretical estimates (introduced later on in Equation (28)).

As evidenced in Figure 9, the localized quasi-degenerated eigenstates below and above the allowed band present different behaviors (see, respectively, at left or right of the gray zone). First, whatever the value of *N*, we observe that the smoothed probability for eigenstates emerging at the top of the band (right side of the gray zone) generally present higher amplitudes than for eigenstates below the allowed band (left side of the gray zone). This is evidenced for N=6, where we see that $P_{max}$ lives in the interval [0.3,0.65] for states below the band, which is smaller than for the state above the band for which $P_{max}\in \left[0.65,0.9\right]$. These intervals tend to slightly change when N=12 to become, respectively, [0.2,0.8] and [0.5,0.9] on the left and right hand of the gray zone. Second, focusing now on the transfer time $T_{max}$ (second row in Figure 9), we clearly see that the latter is generally lower for eigenstates emerging at the top of the band (right side of the gray zone) than for the other ones (left side of the gray zone). Indeed, if we consider two distinct *M* values that give rise to two quite similar $P_{max}$ values, it turns out that the time $T_{max}$ associated with the larger *M* value is always significantly shorter than the time $T_{max}$ associated with the smaller *M* value. For instance, in Figure 9d, for both values M=6 and M=18, the amplitude $P_{max}$ is approximately equal to 0.55. However, $T_{max}=64$ J^−1^ for M=18, whereas it reaches $T_{max}=566$ J^−1^ for M=6. This effect becomes increasingly important the further one moves away from the allowed band.

**Figure 10:**
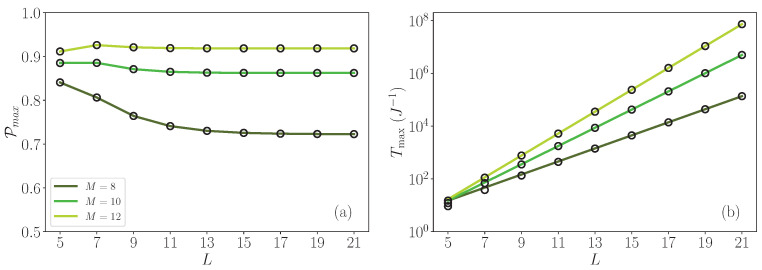
*L* dependence (**a**) of the maximum value of the smoothed probability $P_{max}$ and (**b**) of the time $T_{max}$ for which the maximum arises. The degree of the roots is N=3, and three branching rates have been considered, namely M=8, M=10, and M=12.

These previous results suggest that to optimize the quantum transfer between the two roots, it would be wise to design the architecture of the modular glued trees to favor the occurrence of localized states above the allowed band. However, in this case a fundamental question remains: what is the influence of the graph size on the efficiency of the transfer? To address this question, the *L* dependence of both $P_{max}$ and $T_{max}$ is displayed in Figure 10. The calculations are carried out for N=3, a value for which the spectrum exhibits high-energy localized states above the allowed band, provided that M>7. According to Figure 10a, the amplitude $P_{max}$ becomes less and less sensitive to the size of the graph as *M* increases. For M=8, it slightly decreases with *L*, ranging from 0.84 for L=5 to 0.752 for L=21. By contrast, for M=12, $P_{max}$ is almost *L* independent since it varies from 0.91 for L=5 to 0.92 for L=21. In a marked contrast, Figure 10b reveals that $T_{max}$ behaves in a completely different way. Indeed, $T_{max}$ exhibits an exponential growth with the size of the graph, a behavior that can be enhanced by increasing *M*. Indeed, for L=5 a quite fast transfer occurs since $T_{max}$ varies from 9 J^−1^ for M=8 to 15 J^−1^ for M=12. When L=11, the exponential growth of $T_{max}$ drastically affects the efficiency of the transfer since it varies from 441 J^−1^ for M=8 to 5205 J^−1^ for M=12. This effect becomes dramatic for longer graphs. When L=21, $T_{max}$ varies from $1.36\times 10^{5}$ J^−1^ for M=8 to $7.3\times 10^{7}$ J^−1^ for M=12.

## 4. Interpretation and Discussion

The numerical results reveal that the transfer of a quantum walker between the roots of a modular glued tree strongly depends on the architecture of the graph. As a result, three different regimes were identified, depending on the value of the branching rate, *M*, versus the degree of the roots, *N*. Indeed, as explained in Section 2.2, the behavior of the walker is isomorphic to that of a particle moving on a finite-size chain. This chain exhibits defects that originate in the specific nature of both the roots and the leaves of the glued trees (see Figure 2). The energy spectrum of the chain is particularly rich and three kinds of eigenstates have been identified, giving rise to three different dynamical regimes.

Basically, when *M* is about *N*, the walker exhibits extended states that correspond to superpositions of forward and backward traveling waves whose energies belong to the so-called allowed band. The dynamics is therefore governed by the back-and-forth motion of the initial wave packet between the two roots so that an efficient transfer can take place via quantum recurrences. Such recurrences occur at very precise revival times that may be difficult to detect in an experimental protocol.

Conversely, if *M* strongly differs from *N*, the energy spectrum supports two quasi-degenerated localized states that lie below (if M≪N) or above (if M≫N) the allowed band. These states refer to a localization process on the roots of the glued trees. Consequently, when the walker is initially on the leftmost root, its state preferentially decomposes on these two localized states. The dynamics becomes isomorphic to that of a two-level system, resulting in the occurrence of quantum beats between the two roots. In other words, an almost perfect energy transfer is mediated by these specific localized states. Nevertheless, two distinct regimes arise depending on whether the states emerge below or above the allowed band. Our numerical results suggest that high energy localized states yield a more efficient transfer. In this case, the probability of observing the CTQW on the rightmost root can be very high over a wide time scale, facilitating the experimental detection of the walker.

### 4.1. Eigenstates and Mode Equations

To discuss and interpret the numerical results, let us study the restricted Hamiltonian H that describes the dynamics of the walker in the column subspace. As mentioned above, this Hamiltonian defines a tight-binding model on the finite-size chain depicted on Figure 2. The associated states are thus given by the walker time-independent Schrodinger equation, written as

(12) $$\sum_{s^{\prime }=1}^{L}H_{ss^{\prime }}\phi \left(s^{\prime }\right)=\epsilon \phi \left(s\right).$$

According to the standard properties of finite-size tight-binding models [48,49], the solutions of Equation (12) are superpositions of forward and backward traveling waves with wave vector *q* as

(13) $$\phi \left(s\right)=\left\{\begin{array}{cc} A & \mathrm{if}\;\;s=1\\ A^{\left(+\right)}e^{iqs}+A^{\left(-\right)}e^{-iqs} & \mathrm{if}\;\;1<s<L_{c}\\ C & \mathrm{if}\;\;s=L_{c}\\ B^{\left(+\right)}e^{iqs}+B^{\left(-\right)}e^{-iqs} & \mathrm{if}\;\;L_{c}<s<L\\ B & \mathrm{if}\;\;s=L. \end{array}.\right.$$

By inserting this solution into Equation (12) far from the defects, it turns out that eigenenergies satisfy the dispersion relation of the infinite chain $\epsilon_{q}=\epsilon_{0}+2\Phi \cos\left(q\right)$. However, the value of the wave vector *q* is still unknown at this stage. To determine the allowed wave vector, one proceeds as follows. First, because the chain is symmetric with respect to the central node, the wave function is either symmetric (A=B, $A^{\left(\pm \right)}=B^{\left(\pm \right)}$, C≠0) or anti-symmetric (A=−B, $A^{\left(\pm \right)}=-B^{\left(\pm \right)}$, C=0), and it is characterized by 4 amplitudes. Second, by studying the Schrodinger equation for s=1, s=2, $s=L_{c}-1$, and $s=L_{c}$, one obtains a system of 4 equations for the unknown amplitudes for each symmetry. These two systems exhibit non-trivial solutions if and only if their determinant vanishes. After algebraic manipulations, this condition gives rise to the so-called mode equations, i.e., the equations whose solutions specify the allowed *q* values for each symmetry. The mode equation for symmetric states is defined as

(14) $$\left(\frac{{\bar{\Delta }}_{r}-e^{-iq}+\left(z^{2}-1\right)e^{iq}}{{\bar{\Delta }}_{r}-e^{iq}+\left(z^{2}-1\right)e^{-iq}}\right)\left(\frac{{\bar{\Delta }}_{c}+e^{iq}-e^{-iq}}{{\bar{\Delta }}_{c}+e^{-iq}-e^{iq}}\right)=e^{iq(L-1)},$$

whereas the mode equation for anti-symmetric states is expressed as

(15) $$\left(\frac{{\bar{\Delta }}_{r}-e^{-iq}+\left(z^{2}-1\right)e^{iq}}{{\bar{\Delta }}_{r}-e^{iq}+\left(z^{2}-1\right)e^{-iq}}\right)=e^{iq(L-1)},$$

with ${\bar{\Delta }}_{r}=\Delta_{r}/\Phi =\left(M-N+1\right)/\sqrt{M}$, ${\bar{\Delta }}_{c}=\Delta_{c}/\Phi =\left(M-1\right)/\sqrt{M}$, and $z=\sqrt{N/M}$.

By studying the mode equations, it turns out that the chain supports extended states characterized by a real wave vector *q*. These states define traveling waves whose eigenenergies belong to the energy band $\epsilon_{q}\in \left[\epsilon_{0}-2\Phi ,\epsilon_{0}+2\Phi \right]$. From a physical point of view, they describe states uniformly delocalized over the columns of the glued trees and which are able to propagate between the two roots so that a stationary regime takes place. However, since the chain exhibits defects that break the symmetry of the problem, the Hamiltonian H supports additional eigenstates whose properties strongly differ from those of the traveling waves. These states correspond to wave functions that are localized in the neighborhood of the defects and whose energies lie outside the allowed band. These are thus characterized by a complex wave vector q=iκ (for states lying above the band) or q=π+iκ (for states lying below the band), with κ>0. In that context, to solve the mode equations for the localized states, it is wise to introduce the real variable $x=e^{-iq}$ that satisfies |x|>1. With this notation, the energy of a localized state is defined as $\epsilon =\epsilon_{0}+\Phi \left(x+x^{-1}\right)$ and the mode equations are rewritten as

(16) $$\left\{\begin{array}{cc} F\left(x\right)G\left(x\right)=x^{-(L-1)}F\left(x^{-1}\right)G\left(x^{-1}\right) & \mathrm{for}\;\mathrm{symmetric}\;\mathrm{states}\\ F\left(x\right)=x^{-(L-1)}F\left(x^{-1}\right) & \mathrm{for}\;\mathrm{anti}\text{-}\mathrm{symmetric}\;\mathrm{states}, \end{array}\right.$$

with $F\left(x\right)={\bar{\Delta }}_{r}-x+\left(z^{2}-1\right)x^{-1}$ and $G\left(x\right)={\bar{\Delta }}_{c}+x-x^{-1}$. In the finite-size chain, the mode equations cannot be solved analytically. Nevertheless, they can be used to introduce relevant approximations and consequently to understand the numerical observations, as will be shown in the following. To proceed, the main idea consists of a two-step approach in which one first treats the localization in the limit L→∞. In doing so, it will be shown that each side of the chain exhibits degenerated localized states and that a third localized state arises on the central node of the chain. The second step consists of considering finite *L* values for which a coupling occurs between the different localized states.

### 4.2. Localization in the Limit L→∞

In the limit L→∞, the right-hand side of the mode equation, Equation (16), vanishes for |x|>1. Consequently, localized states in the neighborhood of the roots are characterized by the mode equation F(x)=0, that is

(17) $$x^{2}-{\bar{\Delta }}_{r}x+1-z^{2}=0.$$

Equation (17) holds whatever the symmetry of the states since in the limit L→∞ the two roots are independent. It thus refers to two independent localization processes that arise either on the left root s=1 or on the right root s=L. In that case, since *M* is a positive integer, Equation (17) exhibits only one physically acceptable solution, defined as

(18) $$x_{r}=\frac{M-N}{\sqrt{M}}.$$

Note that the second solution is equal to $1/\sqrt{M}$, a very important detail that will make two situations arise, depending on the value of the parameters. Indeed, if $M>{\left(\sqrt{1/4+N}+1/2\right)}^{2}$, then $x_{r}>1$, indicating that the localized states are characterized by a wave vector q=iκ. They thus correspond to two degenerated energy levels located above the allowed band. Conversely, if $M<{\left(\sqrt{1/4+N}-1/2\right)}^{2}$, then $x_{r}<-1$. The localized states are now characterized by a wave vector q=π+iκ so that they refer to two degenerated energy levels that lie below the allowed band. Whatever the situation, the expression of the energy remains the same and is defined as

(19) $$\epsilon_{r}=-\left(N+1\right)J+\frac{M}{M-N}J.$$

One state, characterized by the wave function $\phi_{L}\left(s\right)$, is exponentially localized near the leftmost root s=1. The second state, whose wave function is denoted $\phi_{R}\left(s\right)$, describes a localization near the rightmost root s=L. These wave functions are defined as

(20) $$\begin{array}{ccc} \phi_{L}\left(s\right) & = & {\left(\frac{x_{r}^{2}-1}{x_{r}^{2}-1+z^{2}}\right)}^{\frac{1}{2}}\left(\delta_{s1}+z\left(1-\delta_{s1}\right)\right)x_{r}^{-(s-1)}\\ \phi_{R}\left(s\right) & = & {\left(\frac{x_{r}^{2}-1}{x_{r}^{2}-1+z^{2}}\right)}^{\frac{1}{2}}\left(\delta_{sL}+z\left(1-\delta_{sL}\right)\right)x_{r}^{-(L-s)}. \end{array}$$

As previously, since the right-hand side of Equation (16) vanishes in the limit L→∞, the localized state in the neighborhood of the central node $s=L_{c}$ is characterized by the mode equation G(x)=0, that is

(21) $$x^{2}-{\bar{\Delta }}_{c}x-1=0.$$

Equation (21) exhibits only one physically acceptable solution defined as $x_{c}=\sqrt{M}$. Therefore, provided that M>1, the chain exhibits a state characterized by a wave vector q=iκ, whose energy lies above the allowed band. This energy is strictly equal to zero, i.e., $\epsilon_{c}=0$. This state is exponentially localized around the central node and its wave function $\phi_{c}\left(s\right)$ is defined as

(22) $$\phi_{c}\left(s\right)={\left(x_{c}^{2}-1\right)}^{\frac{1}{2}}x_{c}^{-|s-L_{c}|}.$$

At this step, let us mention that, for N=6, the previous calculations reveal that states localized on the roots lie above the allowed band for M>9 and below the allowed band for M<4. For M=3, the energy of the localized states is equal to $\epsilon_{r}=-8$ J, whereas for M=15 it reaches $\epsilon_{r}=-5.33$ J. Moreover, whatever *M*, the energy of the state localized on the central node is equal to $\epsilon_{c}=0$. All these results are in very good agreement with the numerical observations reported in Section 3 and displayed in Figure 3.

### 4.3. Localized State-Mediated Quantum Transfer

In a finite-size chain, the localized states are no longer independent and they interact through the overlap of their wave functions. However, since $\epsilon_{c}$ is larger than $\epsilon_{r}$, the preferential interaction is between the states $\phi_{L}$ and $\phi_{R}$, which are localized on the leftmost root and on the rightmost root, respectively. Since these two states enter into resonance, the latter combine and a quantum superposition arises, giving rise to the occurrence of a symmetric state $\phi_{s}\approx \left(\phi_{L}+\phi_{R}\right)/\sqrt{2}$ and of an anti-symmetric state $\phi_{a}\approx \left(\phi_{L}-\phi_{R}\right)/\sqrt{2}$. The formation of these states is accompanied by the rise of degeneracy due to the so-called avoided crossing phenomena. The states $\phi_{s}$ and $\phi_{a}$ are thus characterized by two distinct energies, $\epsilon_{s}$ and $\epsilon_{a}$, which can be calculated from the solutions of the mode equations, Equation (16). However, these mode equations reveal a very important feature. Indeed, for the anti-symmetric state $\phi_{a}$, the mode equation depends on ${\bar{\Delta }}_{r}$ and *z* only. This would imply that the formation of $\phi_{a}$ only results from the quantum superposition between $\phi_{L}$ and $\phi_{R}$. It does not involves the coupling with the state $\phi_{c}$ localized on the central node. Conversely, for the symmetric state, the mode equation involves ${\bar{\Delta }}_{r}$, ${\bar{\Delta }}_{c}$, and *z*. This feature reveals that the origin of $\phi_{s}$ is in fact twofold. First, as mentioned previously, $\phi_{s}$ originates in the quantum superposition between $\phi_{L}$ and $\phi_{R}$. However, a remaining coupling arises with $\phi_{c}$. This coupling is responsible for an additional energy shift that affects the symmetric state, but not the anti-symmetric state. In this context, when states localized on the roots are present, the dynamics of the CTQW can be interpreted as follows. If the walker is initially located on the leftmost root s=1, its behavior is mainly governed by these localized states. The quantum dynamics is thus isomorphic to that of a two-level system formed by the symmetric state $\phi_{s}$ and the anti-symmetric state $\phi_{a}$. Therefore, as a first approximation, the evolution operator U(t) defined in Equation (9) can be expressed in a simpler way, as

(23) $$U\left(t\right)\approx \exp\left(-i\epsilon_{s}t\right)|\phi_{s}\rangle \langle \phi_{s}|+\exp\left(-i\epsilon_{a}t\right)|\phi_{a}\rangle \langle \phi_{a}|.$$

The probability for observing the walker on the rightmost root at time *t* given that it occupies the leftmost root at t=0 is thus defined as

(24) $$P_{L|1}\left(t\right)\approx \frac{1}{2}|\phi_{L}{\left(1\right)|}^{2}{\left|\phi_{R}\left(L\right)\right|}^{2}\left(1-\cos\left(\Delta \epsilon t\right)\right).$$

with $\Delta \epsilon =\epsilon_{s}-\epsilon_{a}$. At this step, the energy difference Δϵ still remains unknown. Its calculation requires one to solve the mode equations, Equation (16), for finite *L* values, a task that cannot be achieved analytically. To overcome this problem, we propose to use an approximate procedure based on an expansion of the mode equations around the solutions in the limit L→∞. To proceed, let us first consider the mode equation for the anti-symmetric state $\phi_{a}$. This equation, defined as $F\left(x\right)=x^{-(L-1)}F\left(x^{-1}\right)$, gives rise to the solution $x_{a}$, which allows us to obtain the corresponding energy $\epsilon_{a}=\epsilon_{0}+\Phi \left(x_{a}+x_{a}^{-1}\right)$. Given that the solution is $x_{r}$ in the limit L→∞ (Equation (18)), one seeks a solution $x_{a}=x_{r}+\delta x_{a}$. By expanding the mode equation around $x_{r}$, one obtains, to the lowest order,

(25) $$\delta x_{a}\approx x_{r}^{-(L-1)}F\left(x_{r}^{-1}\right){\left(\frac{\partial F}{\partial x}\right)}_{x_{r}}^{-1}.$$

For $\phi_{s}$, the mode equation is defined as $F\left(x\right)G\left(x\right)=x^{-(L-1)}F\left(x^{-1}\right)G\left(x^{-1}\right)$. Its solution $x_{s}$ yields the corresponding energy $\epsilon_{s}=\epsilon_{0}+\Phi \left(x_{s}+x_{s}^{-1}\right)$. As previously, by seeking a solution $x_{s}=x_{r}+\delta x_{s}$, one obtains, to the lowest order,

(26) $$\delta x_{s}\approx x_{r}^{-(L-1)}\frac{F\left(x_{r}^{-1}\right)G\left(x_{r}^{-1}\right)}{G(x_{r})}{\left(\frac{\partial F}{\partial x}\right)}_{x_{r}}^{-1}.$$

Therefore, after some algebraic calculations we are able to determine the expression of both $\delta x_{a}$ and $\delta x_{s}$, and to obtain the energy difference Δϵ, written as

(27) $$\Delta \epsilon \approx \frac{2Je^{-\kappa (L-1)}}{M{\left(M-N\right)}^{2}}\left(\frac{{\left[M-{\left(M-N\right)}^{2}\right]}^{3}}{{\left(M-N\right)}^{2}-1}\right),$$

with $\kappa =\ln|x_{r}|$. At this step, let us mention that the energy of the symmetric state localized on the central node is equal to zero whatever *L*. Indeed, $x_{c}=\sqrt{M}$ is a solution of the mode equation $F\left(x\right)G\left(x\right)=x^{-(L-1)}F\left(x^{-1}\right)G\left(x^{-1}\right)$ since $G(x_{c})=0$ (see Equation (21)) and $F(x_{c}^{-1})=0$ (see the note after Equation (18)). Finally, by combining Equations (20), (24), and (27), one obtains an approximate expression for the probability of observing the walker on the rightmost root, as

(28) $$P_{L|1}\left(t\right)\approx \frac{1}{2}P_{max}\left[1-\cos\left(\frac{\pi t}{T_{max}}\right)\right],$$

with $T_{max}=\pi /\Delta \epsilon$ and

(29) $$P_{max}={\left(1+\frac{z^{2}}{\left(x_{r}^{2}-1\right)}\right)}^{-2}={\left(\frac{{\left(M-N\right)}^{2}-M}{{\left(M-N\right)}^{2}-\left(M-N\right)}\right)}^{2}.$$

In a qualitative agreement with the numerical observations, Equation (28) shows that the probability $P_{L|1}\left(t\right)$ is a periodic function whose period is equal to $T=2T_{max}$. Initially equal to zero, it increases to reach its first maximum value $P_{max}$ at time $T_{max}$. As shown in Figure 5 and Figure 7, Equation (28) provides a good estimate of the time evolution of the smoothed probability (orange lines) when the main part of the dynamics is governed by the states localized on the roots. In addition, as illustrated in Figure 9, it allows us to qualitatively understand the behavior of the maximum value $P_{max}$, as well as the behavior of the time $T_{max}$ (orange symbols).

Equation (28) clearly shows the key role played by the localized states for the efficiency of the transfer between the two roots of the graph. Indeed, the stronger the localization, the more efficient the transfer. This feature originates in the weight of the initial state on both $\phi_{a}$ and $\phi_{s}$. This weight increases as $|x_{r}|$ moves away from unity, which favors the increases of the maximum probability $P_{max}$, which tends to unity. Unfortunately, this optimization of $P_{max}$ has a cost since the stronger the localization, the longer the time $T_{max}$. Indeed, $T_{max}$ increases exponentially as the localization is enhanced. Nevertheless, as observed in Section 3, such a negative effect can be softened by using localized states above the allowed band to mediate the transfer. The main reason for this can be understood as follows. When they lie below the allowed band, the creation of $\phi_{a}$ and $\phi_{s}$ mainly results from the quantum superposition between states localized on each root through the overlap of their wave functions. The influence of the state $\phi_{c}$ localized on the central node is negligible. By contrast, when they lie above the allowed band, the mechanism is slightly different. The energy difference with $\phi_{c}$ is reduced so that the coupling between the symmetric states $\phi_{s}$ and $\phi_{c}$ is no longer negligible. This coupling is responsible for an additional contribution of the energy difference, Δϵ, leading to the shortening of $T_{max}$.

## 5. Conclusions

In this paper, a CTQW based on the Laplacian of a modular glued-tree graph is used for studying the ability of a quantum walker to propagate between the roots of the trees. Instead of considering traditional binary trees, we focused our attention here on leafier structures in which the degree of the roots, *N*, differs from the branching rate, *M*, of the other nodes. Therefore, by mapping the problem onto a one-dimensional CTQW, we have shown that the walker behaves as a particle moving on a finite-size chain that exhibits defects. These defects, that result from the specific nature of both the roots and the leaves of the glued trees, yield a rich energy spectrum. Depending on the architecture of the graph, different kinds of eigenstates have been identified, i.e., extended states and states localized either on the roots or on the leaves, giving rise to different dynamical regimes.

Basically, when *M* is about *N*, the energy spectrum exhibits extended states and one state localized on the leaves. Therefore, the walker dynamics are governed by the back-and-forth motion of the initial wave packet between the two roots. An efficient transfer between the two roots can take place via quantum recurrences that occur at very precise revival times, which may be difficult to detect in an experimental protocol. By contrast, when *M* strongly differs from *N*, the energy spectrum supports two additional quasi-degenerated states outside the allowed band that refer to a localization process on the roots. When the walker is initially on the leftmost root, its state preferentially decomposes on these two localized states so that the dynamics become isomorphic to that of a two level-system. Quantum beats occur between the two roots, resulting in an almost perfect transfer mediated by these specific localized states. Nevertheless, we have shown that a more efficient transfer arises when the localized states lie above the allowed band. In this case, the states localized on the roots interact with the states localized on the leaves. This interaction enhances the rise of the quasi-degeneracy, resulting in the shortening of the transfer time. Therefore, the probability of observing the CTQW on the target rightmost root can be very high over a wide time scale, facilitating the experimental detection of the walker.

Our work evidenced that localized state-mediated almost perfect quantum-state transfer between the roots of two glued trees could be achieved with an appropriate design of the graph architecture. These results naturally motivate new questions that could represent interesting starting points for future developments. First it could be wise to investigate what happens in more realistic networks. In that case, the walker does not propagate freely but interacts with the remaining degrees of freedom of the structures. These interactions could favor decoherence processes that may drastically affect the efficiency of the transfer. Second, it would be interesting to see if the realistic implementations of the CTQW will be able to maintain an almost perfect transfer when the inherent presence of disorder favors the stopping of the propagation of the walker due to the so-called Anderson localization. Finally, in the present situation the defects are not independent from each other since they depend on the branching rate, *M*. Therefore, it could be interesting to see what happens in a one dimensional structure with three independent defects located on both the edges and the center of the network. By tuning the central defect judiciously, it could be possible to optimize the transfer between the two edges of the chain owing to the interaction between states localized on the edges and a state localized on the center of the chain.

## Data Availability

The dataset generated and analyzed in the current study is available from the corresponding author upon reasonable request.
